# Genome-wide identification and expression profile analysis of the NAC transcription factor family during abiotic and biotic stress in woodland strawberry

**DOI:** 10.1371/journal.pone.0197892

**Published:** 2018-06-13

**Authors:** He Zhang, Hao Kang, Chulian Su, Yanxiang Qi, Xiaomei Liu, Jinji Pu

**Affiliations:** 1 Environment and Plant Protection Institute, Chinese Academy of Tropical Agricultural Sciences, Key Laboratory of Integrated Pest Management on Tropical Crops, Ministry of Agriculture, Haikou, Hainan, China; 2 Institute of Tropical Agriculture and Forestry, Hainan University, Haikou, China; Jawaharlal Nehru University, INDIA

## Abstract

The NAC transcription factors involved plant development and response to various stress stimuli. However, little information is available concerning the NAC family in the woodland strawberry. Herein, 37 *NAC* genes were identified from the woodland strawberry genome and were classified into 13 groups based on phylogenetic analysis. And further analyses of gene structure and conserved motifs showed closer relationship of them in every subgroup. Quantitative real-time PCR evaluation different tissues revealed distinct spatial expression profiles of the *FvNAC* genes. The comprehensive expression of *FvNAC* genes revealed under abiotic stress (cold, heat, drought, salt), signal molecule treatments (H_2_O_2_, ABA, melatonin, rapamycin), biotic stress (*Colletotrichum gloeosporioides* and *Ralstonia solanacearum*). Expression profiles derived from quantitative real-time PCR suggested that 5 *FvNAC* genes responded dramatically to the various abiotic and biotic stresses, indicating their contribution to abiotic and biotic stresses resistance in woodland strawberry. Interestingly, *FvNAC* genes showed greater extent responded to the cold treatment than other abiotic stress, and H_2_O_2_ exhibited a greater response than ABA, melatonin, and rapamycin. For biotic stresses, 3 *FvNAC* genes were up-regulated during infection with *C*. *gloeosporioides*, while 6 *FvNAC* genes were down-regulated during infection with *R*. *solanacearum*. In conclusion, this study identified candidate *FvNAC* genes to be used for the genetic improvement of abiotic and biotic stress tolerance in woodland strawberry.

## Introduction

Transcription factors containing the highly conserved NAC domain (NAM-ATAF1/2-CUC) in the N-terminal region compromise is one of the largest transcription factors families in plants [1–3]. The C-terminal region that contains the protein binding activity domain is greatly variable and plays an important role in transcriptional regulation [2, 4–7]. The NAC proteins play crucial roles in various aspects of plant growth and development (including the maintenance of the shoot apical meristem, cell division and expansion, nutrient remobilization, secondary cell wall biosynthesis, fiber development, lateral root development, leaf senescence, flower formation, seed development) [1, 3, 8–18], and adaption to the environment [19], such as abiotic stresses [20–29], and response to pathogen infections [6,22,29–33], including *Magnaporthe oryzae* [22,34], *Phytophthora infestans* [35], *Sclerotinia sclerotiorum* [36], and *Colletotrichum graminicola* [33], to name a few.

To date, genome-wide analyses have identified a large number of NAC family members in several species, including 117 *NAC* genes in the model plant *Arabidopsis thaliana* [7], 151 *NAC* genes in *Oryza sativa* [7], 152 *NAC* genes in *Nicotiana tabacum* [37], 163 *NAC* genes in *Populus trichocarpa* [38], 74 *NAC* genes in *Vitis vinifera* [39], 147 putative *NAC* genes in *Setaria italic* [40], 145 *NAC* genes in *Gossypium raimondii* [41], 167 *NAC* genes in *Musa acuminate* [42], 71 *NAC* genes in *Cicer arietinum* [43], 96 *NAC* genes in *Manihot esculenta* [44], 148 *NAC* genes in *Zea mays* [45], 79 *NAC* genes in *Morus notabilis* [46], 82 *NAC* genes in *Cucumis melo* [47], and 104 *NAC* genes in *Solanum lycopersicum* [48]. However, no information is available on the *NAC* family in the rosaceous fruit crop, woodland strawberry (*Fragaria vesca* L.).

Notably, accumulated evidence has confirmed that a large number of *NAC* genes induced by abiotic stresses play critical roles in the regulation of plant tolerance to abiotic stress. Three *Arabidopsis NAC* genes (*ANAC019*, *ANAC055* and *ANAC072*) showed up-regulation after drought, high salinity, and abscisic acid (ABA) treatments, and positively regulate drought tolerance in the overexpression plants [20]. Similarly, overexpression of *ATAF1* in *Arabidopsis* enhanced plant tolerance to drought, ABA, salt, and oxidative stress [19]. While, *ATAF1* was negatively regulate the defense response to necrotrophic fungi (*Alternaria brassicicola* and *Botrytis cinerea*) and bacterial pathogens (*Pseudomonas syringae* pv. *tomato*) [29]. The same function of *NAC* genes, in increasing the tolerance of plants to abiotic stress, was also found in rice. *SNAC1*, an *NAC* gene from *O*. *sativa*, can improve drought and salt tolerance in rice, and transgenic plants were found to produce a 22–34% higher yield than the control in the field under severe drought stress conditions [21]. Accordingly, *OsNAC10*-overexpressing rice plants showed increased grain yield in comparison to the controls under both normal and drought conditions [23]. Overexpression of 3 rice drought-responsive *NAC* genes (*OsNAC5*, *OsNAC6*, and *OsNAC10*) increased plant tolerance to drought and salt stresses [23,49], and the overexpression of *OsNAC6* led to increased resistance towards rice blast [22,34]. The genes *ZmNAC41* and *ZmNAC100* were found to be induced in *Z*. *mays* during infection with *C*. *graminicola* [33]. Similarly, *BnNAC1-1*, *BnNAC5-1*, and *BnNAC5-7* were induced in *Brassica campestris* during infection with *S*. *sclerotiorum*. NAC family genes thus appear to be crucial regulators of plant tolerance to abiotic and biotic stress, as well as crop yield.

The cultivated strawberry (*Fragaria* × *ananassa* Duch.) is one of the most important fruit crops in the world. The woodland strawberry (*F*. *vesca*, 2n = 2x = 14) has a smaller genome (~ 240 Mb) that is highly congenic with the cultivated strawberry [50]. Robust *in vitro* regeneration and transformation systems have been established for studying the counterparts of many important genes in rosaceous fruit crops [50–53].

Based on the significance of *NACs* in the regulation of plant growth, development and adaption to the environment, the NAC family was selected for systematic analysis in woodland strawberry. In the present study, we identified 37 *NAC* genes from woodland strawberry and performed a detailed investigation of their phylogeny, conserved motifs, gene structure, expression profiles in various tissues and in response to cold, heat, drought, salt stress, signaling of H_2_O_2_, melatonin, ABA and rapamycin, and response to the pathogens *Colletotrichum gloeosporioides* and *Ralstonia solanacearum*. Our results should provide a basis for future research on the evolutionary mechanisms and biotic and abiotic stress responses mediated by NACs in the woodland strawberry.

## Materials and methods

### Plant materials and treatments

Woodland strawberry (*F*. *vesca* L., 2n = 2x = 14) seedlings were cultivated in a growth chamber at 24±2°C under a 14 h/10 h light/dark photoperiod with 80% relative humidity. The *F*. *vesca* seeds collected from Beimu Experimental Station, Yumin County, Xinjiang Province of China. Hundred-day-old woodland strawberry tissues (leaf, stem, root, flower, full reddening fruit) were collected from the greenhouse for the treatments. The cold and heat stress treatments was performed by transferring the plants to a low temperature (4°C) for 48 h following recovery, or to a high temperature (40°C) for 4 h following recovery. The leaves of the plants treated with cold and heat were then collected at 2 h, 6 h, 12 h, 2 d, 7 d, 14 d post-treatment (dpt). Drought or salt stresses were simulated by irrigating potted strawberry plants with 200 mM mannitol, 100 mM NaCl, respectively. The leaves of the plants treated with drought and salt were collected at 2 h, 6 h, 3 d, 14 d, 18 d, 24 d post-treatment. Signal molecule treatments were performed by spraying the woodland strawberry leaves with a solution containing 10 mM H_2_O_2_, 0.1 mM ABA, 0.5 mM melatonin, 0.01 mM rapamycin, and the leaves were collected at 2 h, 6 h, 12 h, 1 d, 2 d, 3 d post-treatment. The control woodland strawberry seedlings were similarly irrigating or spray with distilled water. 1×10^6^ conidiospores/mL *C*. *gloeosporioides* inoculation leaves were collected at 12 h, 22 h, 40 h, 60 h, 96 h, 120 h post-inoculation (hpi), 1×10^8^ CFU *R*. *solanacearum* irrigating woodland strawberry seedlings were collected at 2 h, 6 h, 12 h, 24 h,48 h, 72 h post-inoculation (hpi), and uninfected leaves served as a negative control. All materials harvested from each treatment were immediately frozen in liquid nitrogen and stored at -70°C before for RNA isolation.

### Identification and phylogenetic analyses of the NAC gene family in the woodland strawberry

Whole protein sequences of woodland strawberry were obtained from the Plant Transcription Factor Database version 4.0 (PlantTFDB; http://planttfdb.cbi.pku.edu.cn/) [54], Phytozome version 12.0 (https://phytozome.jgi.doe.gov/pz/portal.html) and Pfam databases (http://pfam.xfam.org/) [55]. The *Arabidopsis* (*Arabidopsis thaliana*) NAC amino acid sequences were acquired from PlantTFDB version 4.0. The rice (*Oryza sativa* subsp. *japonica*) NAC amino acid sequences were acquired from PhantTFDB and Nuruzzaman et al. [7]. Additionally, all the *Arabidopsis* and rice NACs were entered as queries in BLAST and were used to identify the predicted NACs in the woodland strawberry database. The full-length amino acid sequences of the NAC proteins from *Arabidopsis*, rice and woodland strawberry were used to generate a phylogenetic tree based on ClustalX 2.0 alignment [56] and the unrooted neighbor-joining (NJ) method with 1,000 bootstrap replicates in MEGA 6.06 [57].

### Protein properties and sequence analyses

The molecular weight and isoelectric points (pI) of the presumed FvNACs were predicted using the online ExPASy proteomics server database (http://web.expasy.org/compute_pi/)[58]. Information for each *FvNAC* was retrieved from the *F*. *vesca* version 1.1 database. The *FvNAC* genomic sequences and CDS sequences extracted from PlantTFDB were compared using the online GSDS 2.0 software (http://gsds.cbi.pku.edu.cn/) [59] to infer the exon/intron organization of the *FvNAC* genes. The conserved motifs of the *FvNAC* genes were obtained from the online Multiple Expectation Maximization for Motif Elicitation (MEME) (Suite version 4.11.2; http://meme-suite.org/tools/meme).

### Quantitative RT-PCR evaluation of retrotransposon expression

Total RNA was isolated using an RNAprep Pure Plant Kit (TIANGEN, China, Cat. DP441) and the concentration and purity were evaluated by NanoDrop 2000c (Thermo Scientific, USA). Reverse transcription was implemented using 1 μg total RNA of each sample with a FastQuant RT Kit (With gDNase; TIANGEN, China, Cat. KR106-01). Expression of *FvNAC* genes by quantitative real-time PCR (qRT-PCR) analysis, performed in an Applied Biosystems QuantStudio^TM^ 6 Flex Real-Time PCR System using the UltraSYBR mixture from Beijing CoWin Biotech (Cat. CW2601M). The reaction mixture was as follows: 1 μL cDNA template, 10μL 2 × PCR Mix, 0.5μL forward and reverse primers each (10μM), and 8μL ddH_2_O. PCR reactions were carried out with a denaturing step (95°C 10 min) and 40 cycles of denaturing at 95°C for 10 s, followed by annealing at 56°C for 30 s, and elongation at 72°C for 32 s. Primers with high specificity and efficient amplification on the basis of a dissociation curve analysis and agarose gel electrophoresis were used to conduct the quantification analysis. The relative expressions of the target genes were determined using the 2^–ΔΔCt^ method [60]. *Actin-7* gene (LOC101313051) was used as an internal reference gene for all the quantitative real-time PCR analyses in this study [61]. The primers used for qRT-PCR are listed in S1 Table. All experiments were conducted three times with three biological replicates for qRT-PCR analyses. Relative expression levels greater than 2-fold (2-fold higher than control) were considered up-regulated, whereas relative expression levels that were expressed less than 0.5 fold (2-fold lower than control) were considered down-regulated.

## Results

### Genome-wide identification of NACs in the woodland strawberry

A total of 37 *FvNAC* genes were identified in the woodland strawberry genome as putative members of the NAC family from PlantTFDB v4.0 and Phytozome v12, and were designated as *FvNAC01*-*FvNAC37*. The characteristic parameters of all predicted FvNAC proteins are listed in S2 Table, including chromosome location, protein length, molecular weight, theoretical pI and intron numbers. The 37 FvNAC proteins ranged from 64 (FvNAC23) to 1, 317 (FvNAC02) amino acid residues with an average of 426.3 aa, and the pIs ranged from 4.53 (FvNAC16) to 9.40 (FvNAC32) with 25 members showing pI <7, 67.57% and others, pI >7, 32.43% (S2 Table).

### Phylogenetic analysis of woodland strawberry NACs

**Fig 1:**
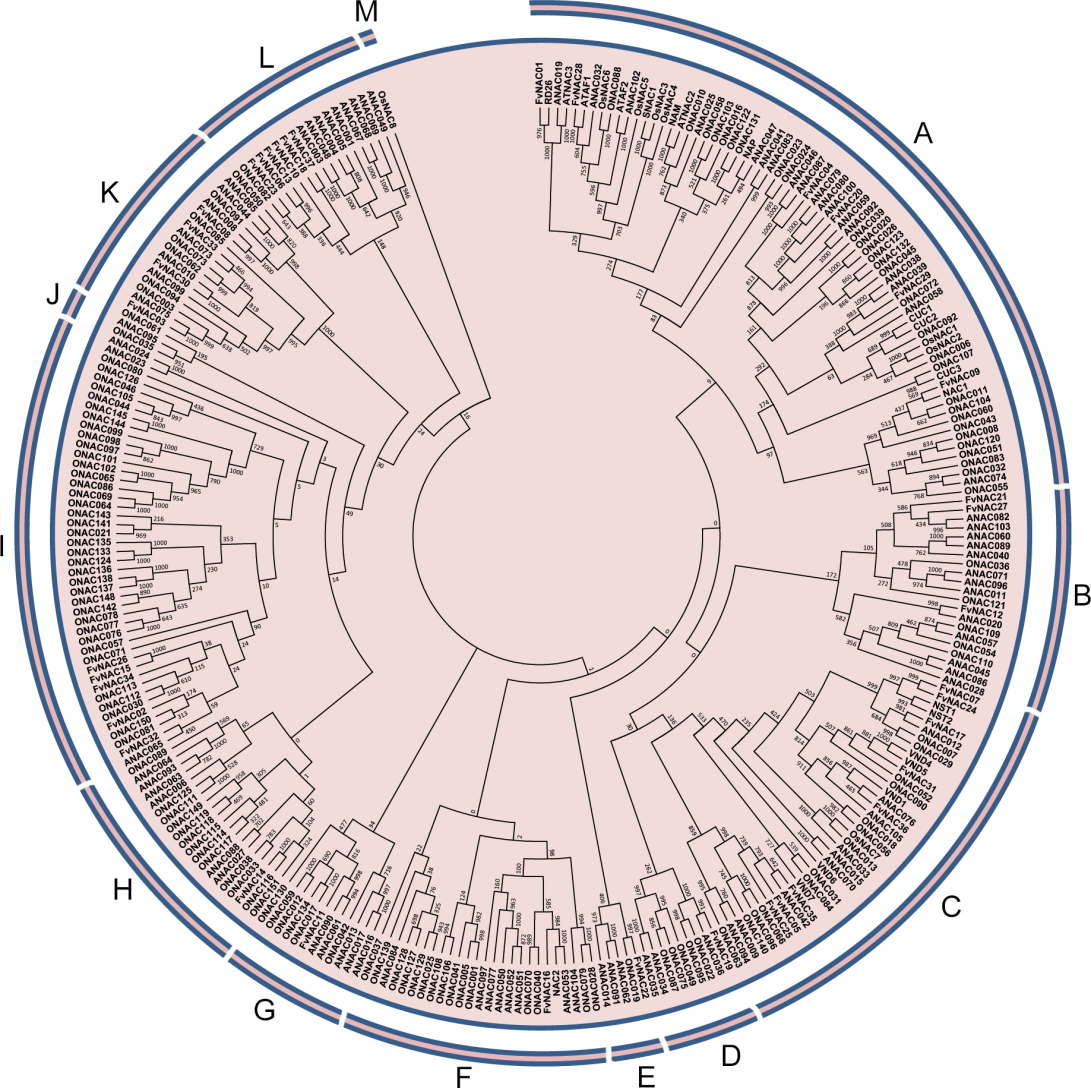
Phylogenetic analysis of NAC proteins from woodland strawberry, rice, and *Arabidopsis*. The full-length amino acid sequences of NAC genes from woodland strawberry (FvNACs), *Arabidopsis* (ANACs), and rice (ONACs) were aligned using ClustalX 2.0, and the phylogenetic tree was constructed using the neighbor-joining method with 1000 bootstrap replicates with MEGA 6.06.

To study the evolutionary relationships between woodland strawberry NAC proteins and known NACs from *Arabidopsis* and rice, an unrooted neighbor-joining phylogenetic tree was constructed with the amino acid sequences of the NAC family proteins from the woodland strawberry, *Arabidopsis*, and rice. The results indicated that 37 FvNACs could be divided into 13 groups (A to M) together with their orthologs of *Arabidopsis* and rice (Fig 1, S3 Table). Phylogenetic analysis also showed that there were some closely related orthologous NACs between the woodland strawberry and *Arabidopsis* (FvNAC01 and RD26, FvNAC28 and ATAF1, FvNAC09 and NAC1, FvNAC35 and ANAC042, FvNAC19 and ANAC035, FvNAC32 and ANAC065, FvNAC03 and ANAC076, FvNAC30 and ANAC010, FvNAC33 and ANAC073, FvNAC08 and ANAC008) and between the woodland strawberry and rice (FvNAC22 and ONAC019, FvNAC14 and ONAC116, FvNAC23 and ONAC082), suggesting that FvNACs are closely associated with the proteins from *Arabidopsis*.

### Gene structure and conserved motifs of woodland strawberry NACs

**Fig 2:**
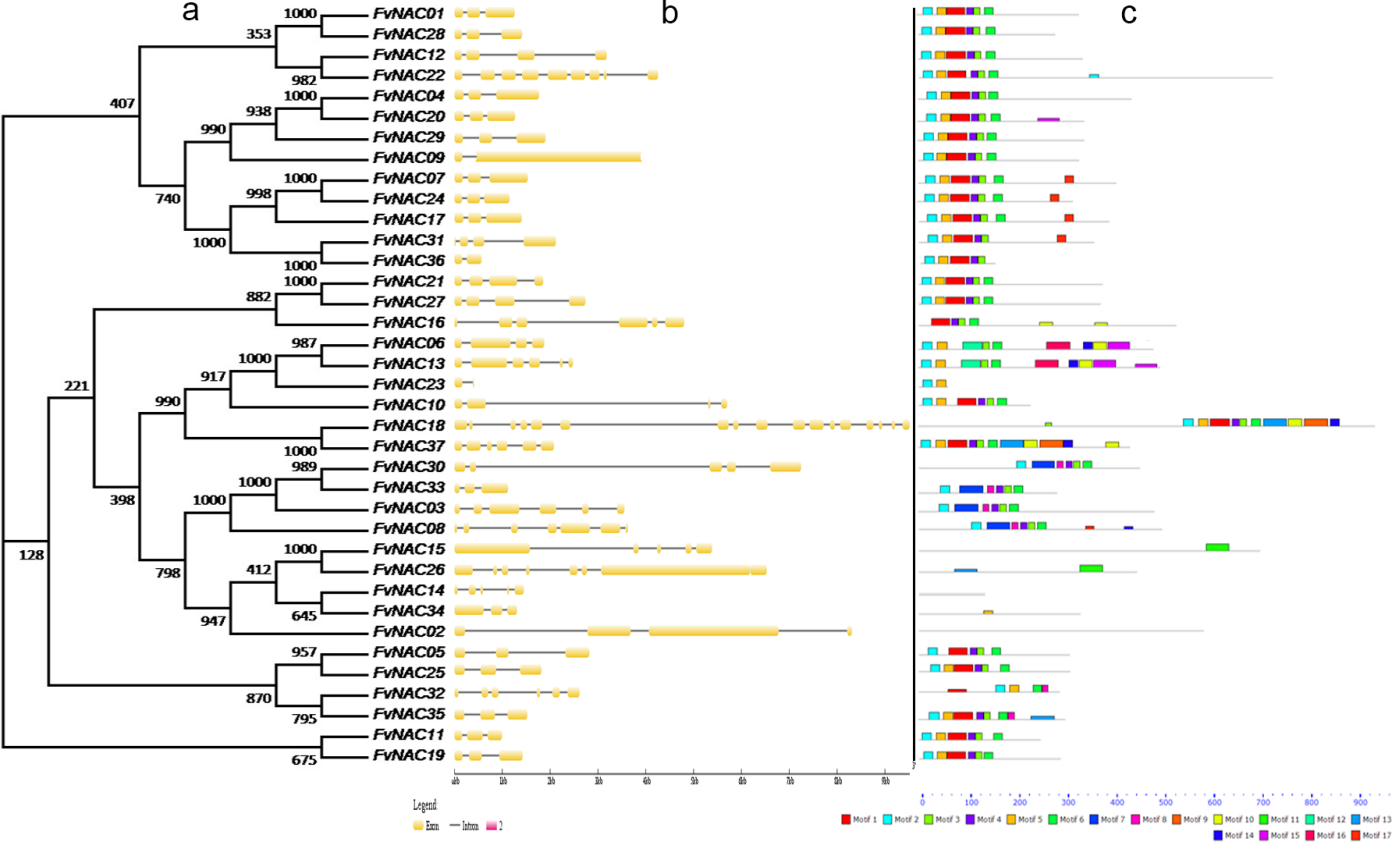
Phylogenetic relationships, exon-intron structure, and motif compositions of *FvNAC* genes. (a) The unrooted phylogenetic tree was constructed using full-length protein sequences of 37 *FvNAC* genes by the neighbor-joining method using 1000 bootstrap replicates. (b) Exon-intron structure analyses of *FvNAC* genes were performed by using the online tool GSDS 2.0. Lengths of exons and introns of each FvNAC genes were exhibited proportionally. (c) The motif composition related to each FvNAC protein. The motifs numbered 1–17 are displayed in different colored boxes. The sequence information for each motif is provided in S1 Fig.

The diversification of gene structure during the evolution of multigene families facilitated the evolutionary co-option of genes for new functions in order to adapt to changes in the environment. To further examine the structural features of *FvNAC* genes, intron/exon distribution and conserved motifs were analyzed according to their phylogenetic relationships (Fig 2A). Gene structure analysis (Fig 2B) indicated that the number of introns of *FvNACs* varied from 1 (*FvNAC23*, *FvNAC36*) to 16 (*FvNAC18*), and it was found that 16 of the 37 *FvNAC* genes had 2 introns, while 7 of the 37 *FvNAC* genes had 3 introns. Most of the *FvNAC* members in the same group exhibited similar exon-intron structure. The conserved intron numbers in each subfamily supports their close evolutionary relationship and group classification.

To further examine the structural diversity of FvNACs, 17 conserved motifs were predicted using Multiple Em for Motif Elicitation (MEME) and subsequent annotation with InterPro (Fig 2C, S1 Fig). The *FvNACs* identified in this study possessed conserved features of the NAC family. Interestingly, it was found that most of the conserved motifs were located in the N-terminal of the NAC proteins that are highly conserved for DNA-binding, indicating that these motifs may be essential for the functioning of NAC proteins. However, the FvNAC18 motifs were located in the C-terminal. FvNAC02 and FvNAC14, lacking motif, and FvNAC34 and FvNAC15, with only a domain, were excluded from further analysis as they cannot be used to construct acceptable phylogenies. In general, the NAC proteins clustered into the same groups and shared similar motif compositions, indicating functional similarities among members of the same group.

### *FvNACs* expression profiles in different organs

**Fig 3:**
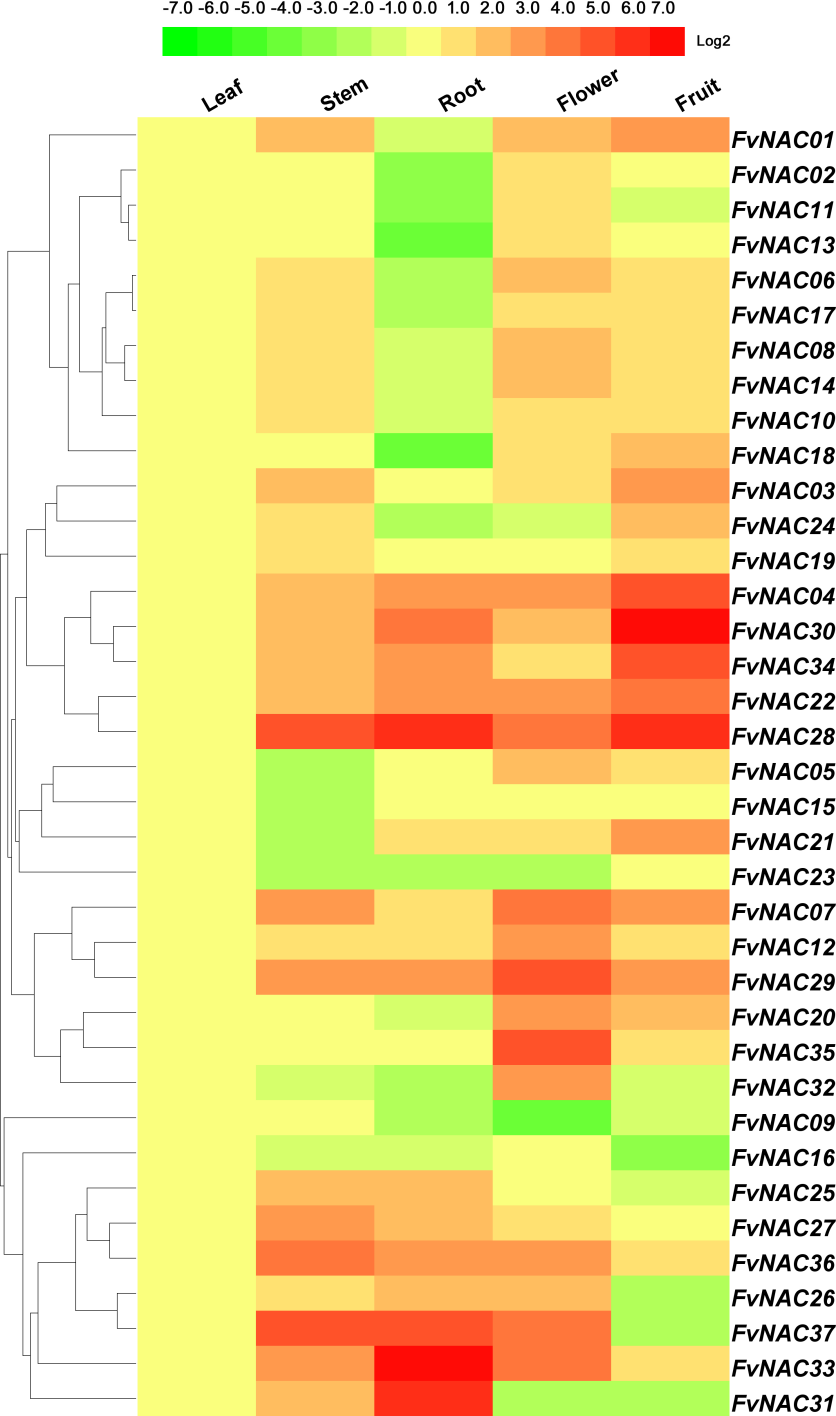
Expression profiles of *FvNAC* genes in different organs of the woodland strawberry. The expression profiles were generated by qRT-PCR and visualized as heat map. The heat map was constructed using HemI 1.0 software. The color scale represents log2 expression values, with green indicates low expression and red indicates high expression.

To determine the biological roles of *NACs* in the woodland strawberry, the distribution of the 37 *FvNACs* transcripts were surveyed in 5 major organs (leaf, stem, root, flower, full reddening fruit) under non-stress conditions. As shown in Fig 3, 10 *FvNAC* genes (*FvNAC-04*, *-07*, *-12*, *-22*, *-28*, *-29*, *-30*, *-33*, *-34*, and *-35*) showed lower transcript abundance in leaves than in the stems, roots, flowers, and full reddening fruit. All 37 *FvNAC* genes showed transcripts in different tissues, in which 24 (64.9%), 16 (43.2%), 29 (78.4%), and 23 (62.1%) genes exhibited high transcriptional abundance (value >2-fold) in the stems, roots, flowers, and full reddening fruit tissues, respectively. *FvNAC13* and *FvNAC18* did not show any transcripts in the roots (S4 Table).

### *FvNACs* expression profiles upon exposure to cold, heat, drought and salt

**Fig 4:**
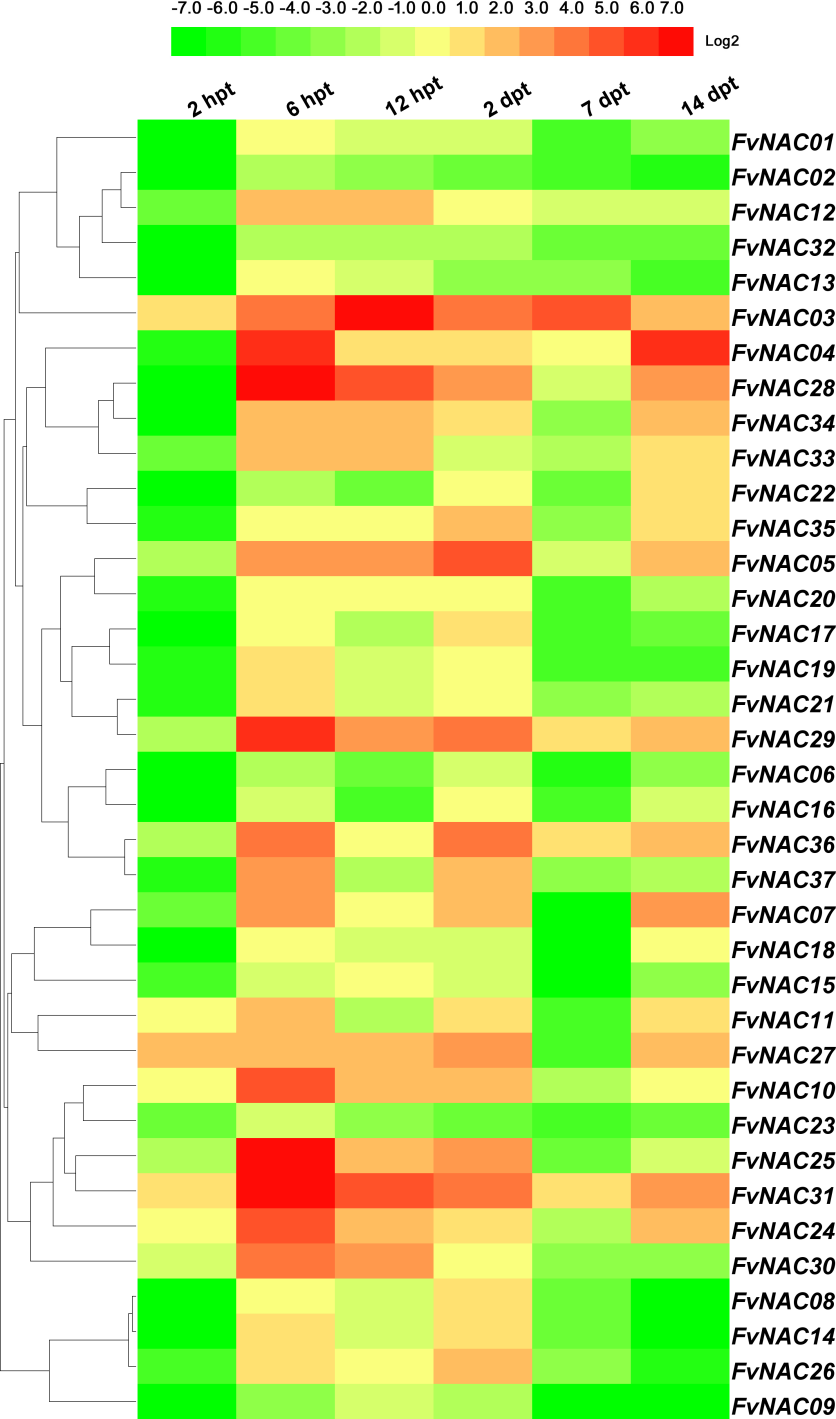
Expression profiles of *FvNAC* genes in leaves under cold stress. The cold stress treatment was performed by transferring the plants to a low temperature (4°C) for 48 h following recovery. Log2 based values from cold stress of qRT-PCR data were used to create the heat map.

**Fig 5:**
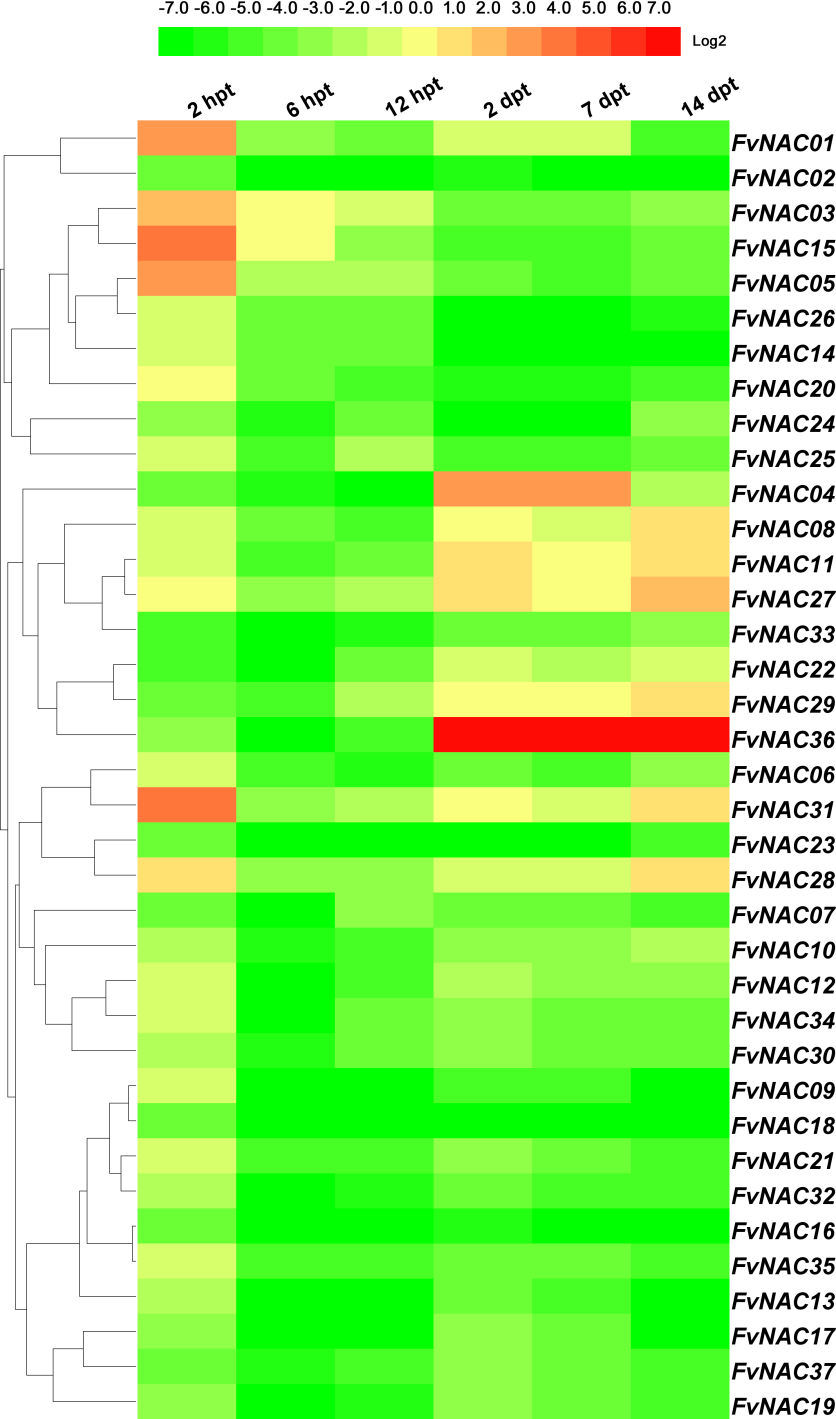
Expression profiles of *FvNAC* genes in leaves under heat stress. The heat stress treatment was performed by transferring the plants to a high temperature (40°C) for 4 h following recovery. Log2 based values from cold stress of qRT-PCR data were used to create the heat map.

**Fig 6:**
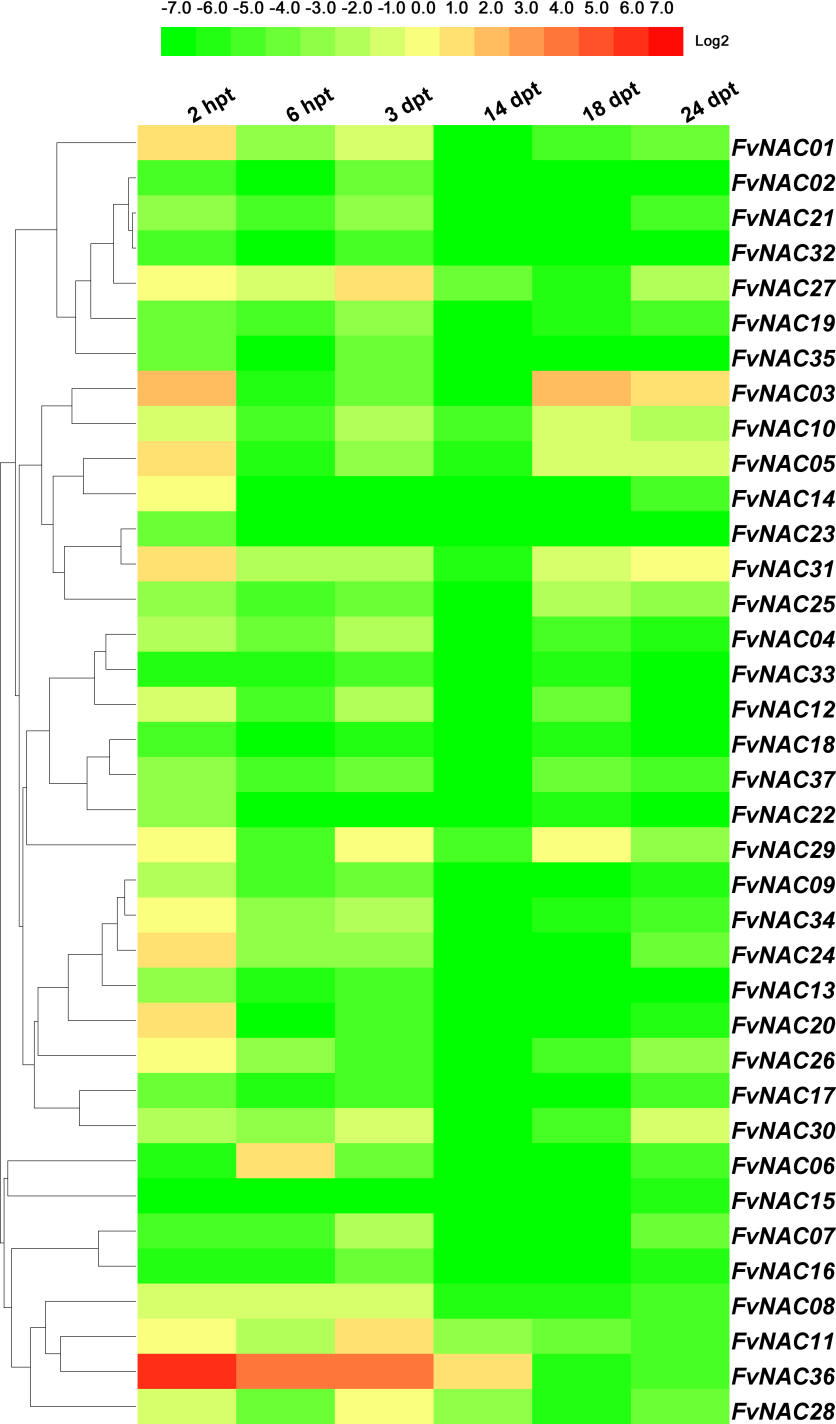
Expression profiles of *FvNAC* genes in leaves under drought stress. The drought stress treatment was simulated by irrigating potted woodland strawberry plants with 200 mM mannitol. Log2 based values from cold stress of qRT-PCR data were used to create the heat map.

**Fig 7:**
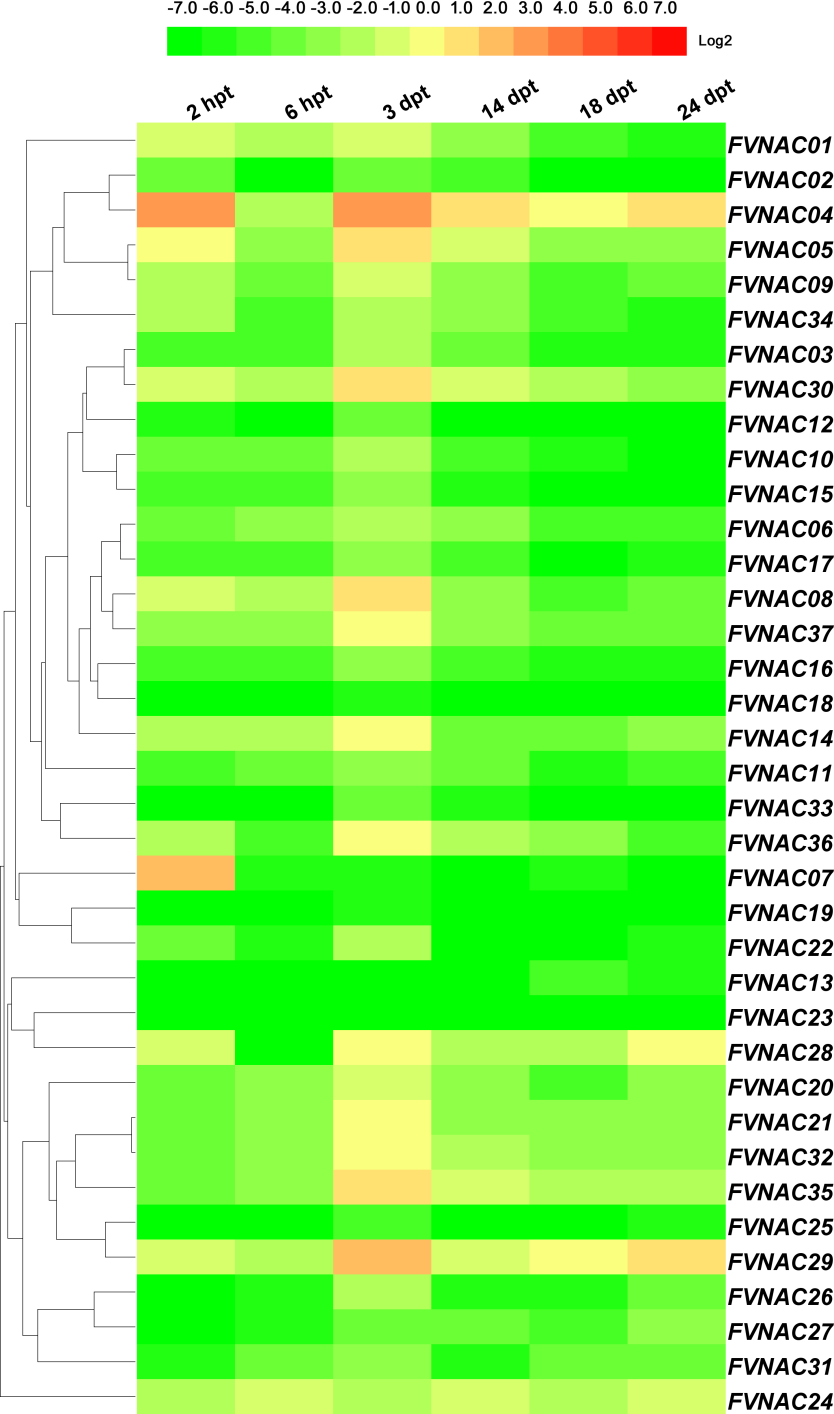
Expression profiles of *FvNAC* genes in leaves under salt stress. The salt stress treatment was simulated by irrigating potted woodland strawberry plants with 100 mM NaCl. Log2 based values from cold stress of qRT-PCR data were used to create the heat map.

To examine the response of *FvNAC* genes to cold (4°C), heat (40°C), drought, and salt stresses at the transcriptional level, the transcripts of *FvNAC* genes under these treatments were determined using quantitative real-time PCR. As shown in Fig 4 (S5 Table), the steady expression of 6 *FvNAC* genes under cold stress at all treated time points was observed, with *FvNAC03* and *FvNAC31* being up-regulated, and *FvNAC02*, *FvNAC06*, *FvNAC09*, and *FvNAC32* being down-regulated. As shown in Fig 5 (S6 Table), 23 *FvNAC* genes (*FvNAC-02*, *-06*, *-07*, *-09*, *-10*, *-12*, *-13*, *-14*, *-16*, *-17*, *-18*, *-19*, *-21*, *-22*, *-23*, *-24*, *-25*, *-30*, *-32*, *-33*, *-34*, *-35*, and *-37*) were down-regulated under heat stress, while no *FvNAC* genes were up-regulated. As shown in Fig 6 (S7 Table), 21 *FvNAC* genes (*FvNAC-02*, *-04*, *-07*, *-08*, *-09*, *-10*, *-12*, *-13*, *-15*, *-16*, *-17*, *-18*, *-19*, *-21*, *-22*, *-23*, *-32*, *-33*, *-35*, and *-37*) were down-regulated under conditions of drought stress. Additionally, as shown in Fig 7 (S8 Table), *FvNAC04* and *FvNAC29* were mostly up-regulated under salt stress, while 20 *FvNAC* genes (*FvNAC-02*, *-03*, *-06*, *-10*, *-11*, *-12*, *-13*, *-15*, *-16*, *-17*, *-18*, *-19*, *-22*, *-23*, *-25*, *-26*, *-27*, *-31*, *-33*, and *-34*) were mostly down-regulated.

### *FvNACs* expression profiles upon exposure to H_2_O_2_, ABA, melatonin and rapamycin

**Fig 8:**
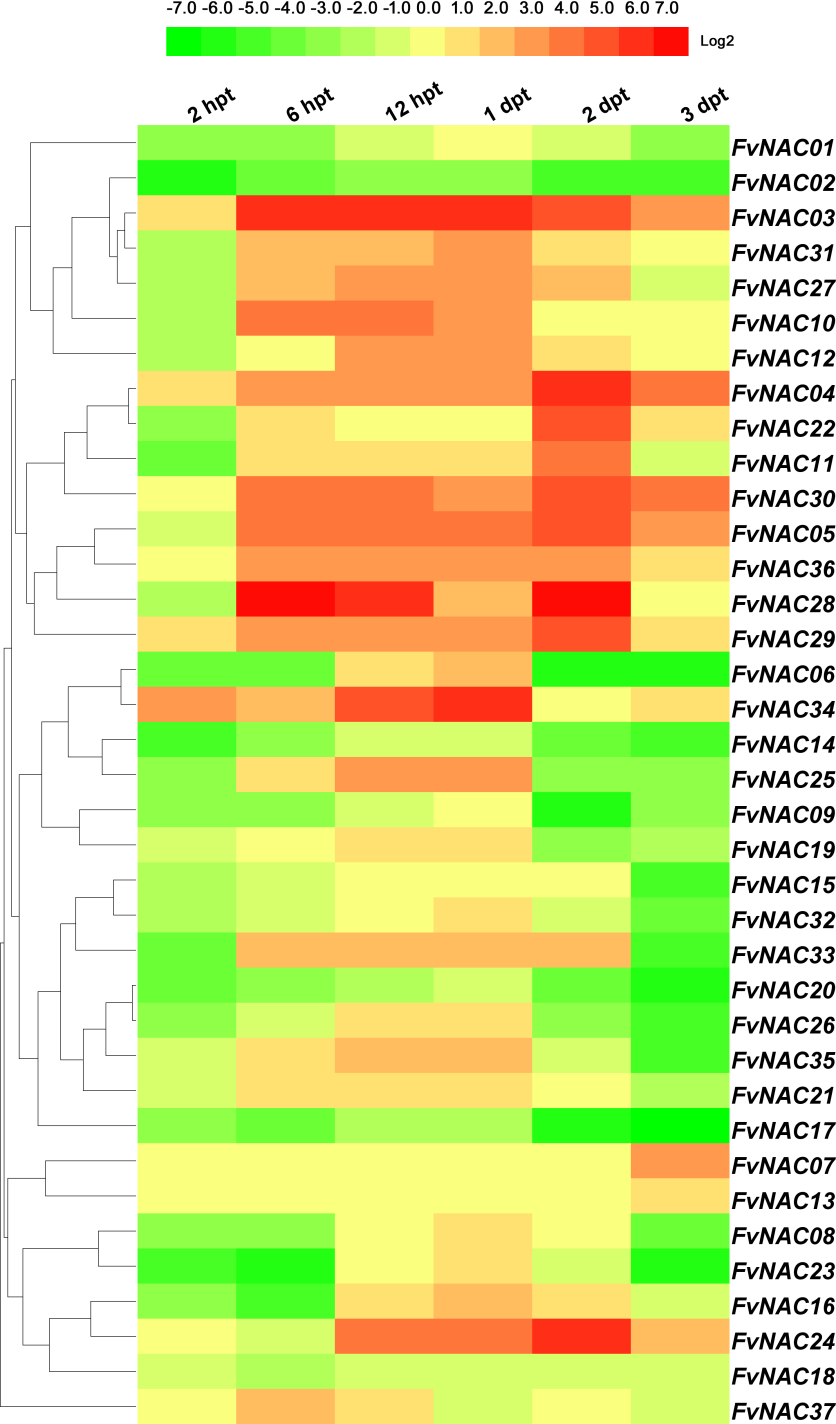
Expression profiles of *FvNAC* genes in leaves under H_2_O_2_ stress. The H_2_O_2_ stress treatment was performed by spraying the woodland strawberry leaves with a solution containing 10 mM H_2_O_2_. Log2 based values from cold stress of qRT-PCR data were used to create the heat map.

**Fig 9:**
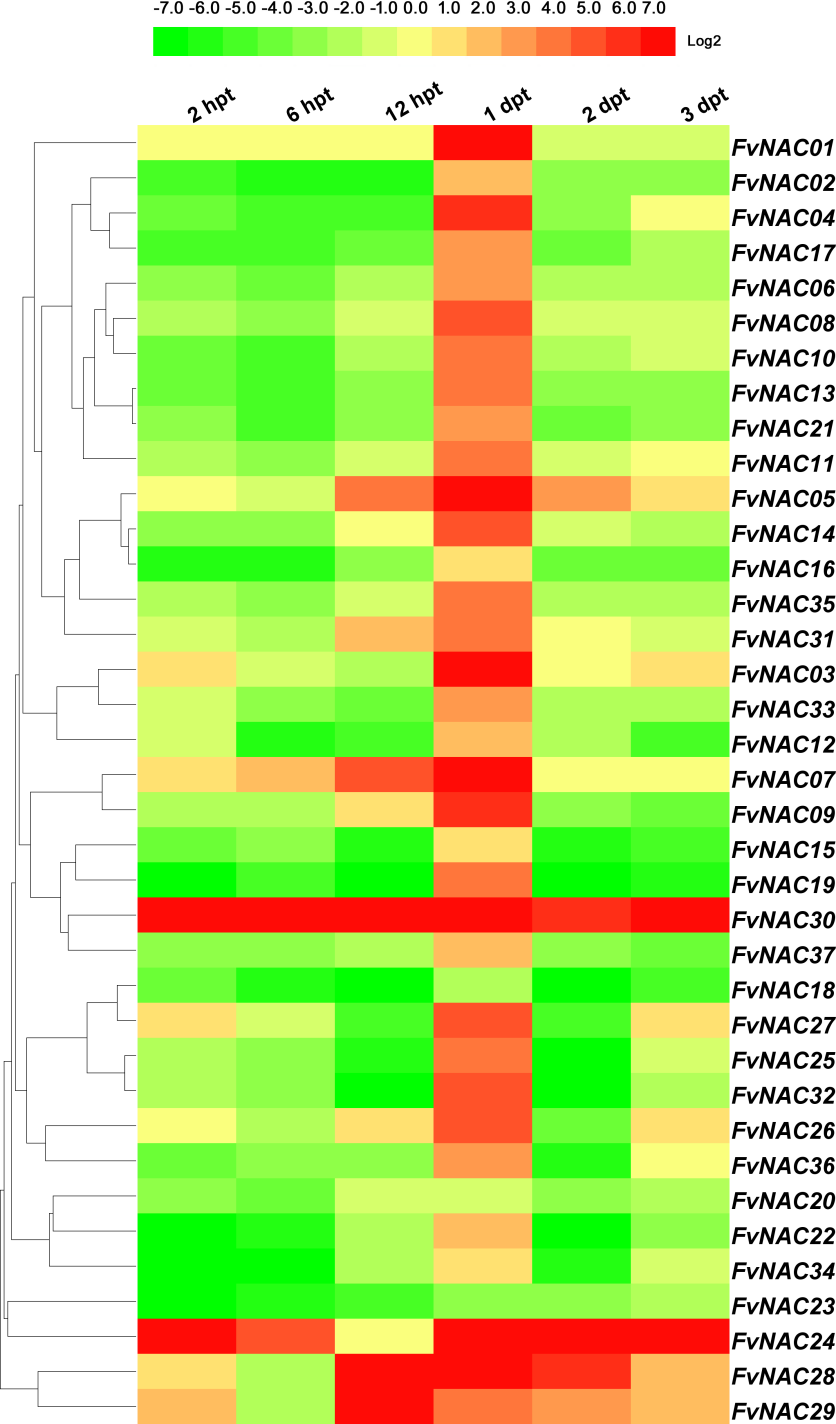
Expression profiles of *FvNAC* genes in leaves under ABA stress. The ABA stress treatment was performed by spraying the woodland strawberry leaves with a solution containing 0.1 mM ABA. Log2 based values from cold stress of qRT-PCR data were used to create the heat map.

**Fig 10:**
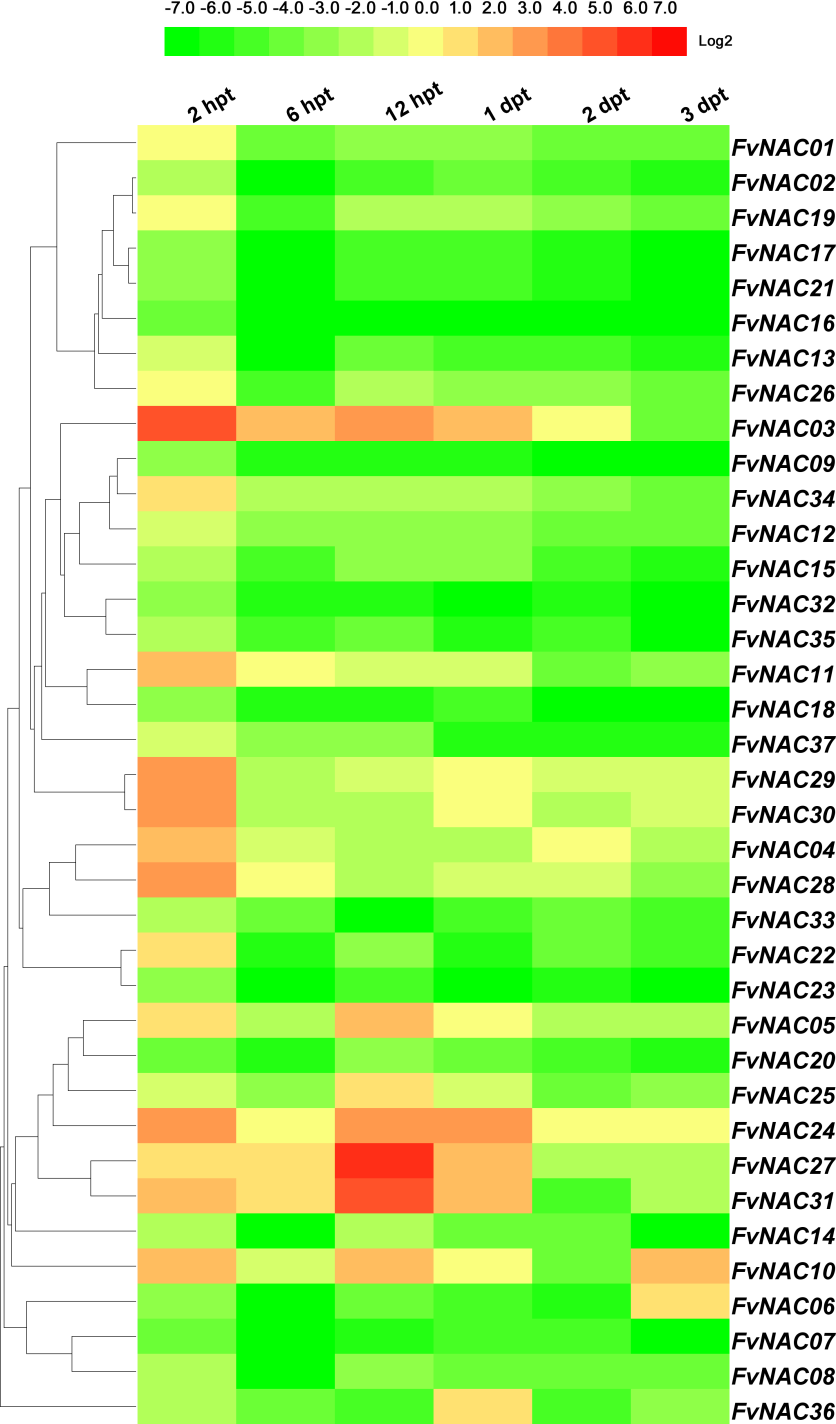
Expression profiles of *FvNAC* genes in leaves under melatonin stress. The melatonin stress treatment was performed by spraying the woodland strawberry leaves with a solution containing 0.5 mM melatonin. Log2 based values from cold stress of qRT-PCR data were used to create the heat map.

**Fig 11:**
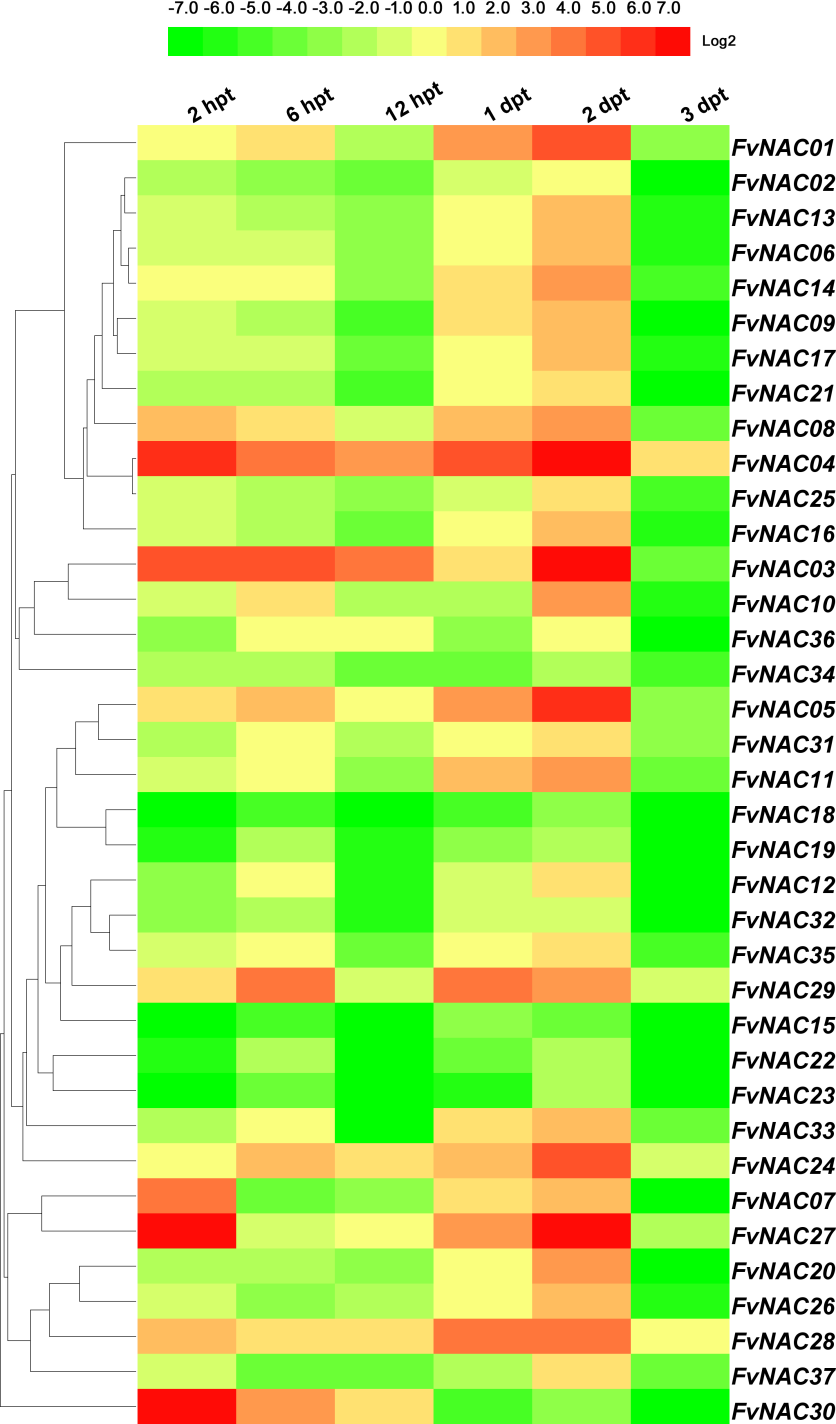
Expression profiles of *FvNAC* genes in leaves under rapamycin stress. The rapamycin stress treatment was performed by spraying the woodland strawberry leaves with a solution containing 0.01 mM rapamycin. Log2 based values from cold stress of qRT-PCR data were used to create the heat map.

To examine the response of *FvNAC* genes to H_2_O_2_, ABA, melatonin, and rapamycin stress at the transcriptional level, the transcripts of the *FvNAC* genes under these treatments were determined by quantitative real-time PCR. As shown in Fig 8 (S9 Table), the steady expression of 7 *FvNAC* genes was observed under H_2_O_2_ stress, with *FvNAC03*, *FvNAC04* and *FvNAC29* being up-regulated, and *FvNAC02*, *FvNAC14*, *FvNAC17* and *FvNAC20* being down-regulated. As shown in Fig 9 (S10 Table), the steady expression of 4 *FvNAC* genes was documented under ABA stress, with *FvNAC30* being up-regulated, and *FvNAC18*, *FvNAC20* and *FvNAC23* being down-regulated. As shown in Fig 10 (S11 Table), 18 *FvNAC* genes (*FvNAC-02*, *-07*, *-08*, *-09*, *-12*, *-13*, *-14*, *-15*, *-16*, *-17*, *-18*, *-20*, *-21*, *-23*, *-32*, *-33*, *-35* and *-37*) were down-regulated under melatonin stress, while no *FvNAC* genes were up-regulated. Moreover, as shown in Fig 11 (S12 Table), the steady expression of 8 *FvNAC* genes was observed under rapamycin stress, with *FvNAC04* being up-regulated, and *FvNAC15*, *FvNAC18*, *FvNAC19*, *FvNAC22*, *FvNAC23*, *FvNAC32* and *FvNAC34* being down-regulated. Notably, *FvNAC18* tended to be down-regulated under all 4 stress treatments.

### *FvNACs* expression profiles in response to *C*. *gloeosporioides* and *R*. *solanacearum* infection

**Fig 12:**
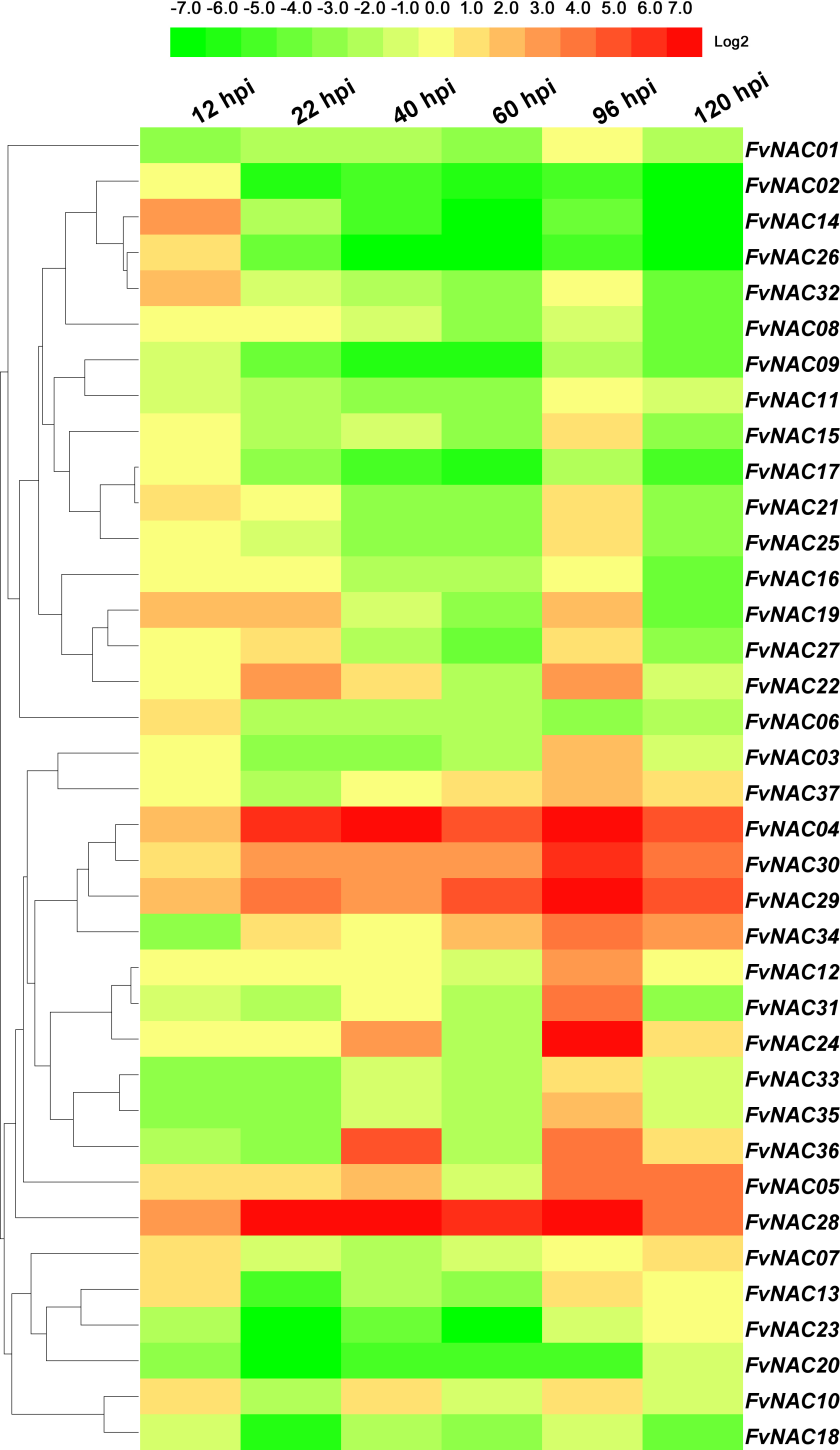
Expression profiles of *FvNAC* genes in leaves under *C. gloeosporioides* infection. The *C. gloeosporioides* infection stress was performed by spraying the conidiospores (1×106 conidiospores/mL) to woodland strawberry leaves surface. Log2 based values from cold stress of qRT-PCR data were used to create the heat map.

**Fig 13:**
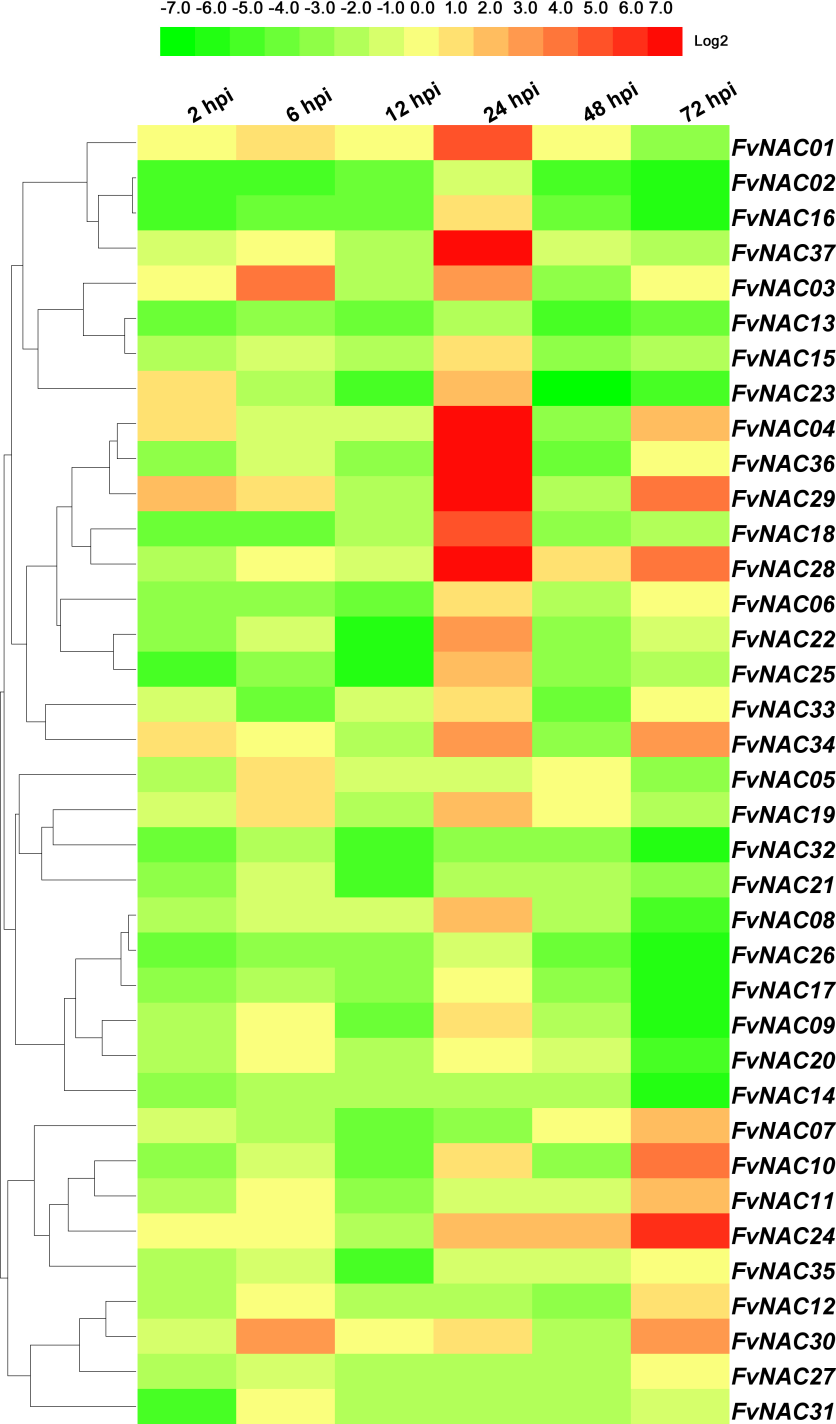
Expression profiles of *FvNAC* genes in leaves under *R. solanacearum* infection. The *R. solanacearum* infection stress was irrigating pathogenic bacteria suspension (1×10^8^ CFU) woodland strawberry seedlings.

To investigate the possible role of *FvNAC* genes in plant-pathogen interactions, quantitative real-time PCR was used to analyze the response of woodland strawberry leaf infected with *C*. *gloeosporioides* and *R*. *solanacearum* in comparison to a control. As shown in Fig 12 (S13 Table), the steady expression of 6 *FvNAC* genes occurred under *C*. *gloeosporioides* infection, with *FvNAC04*, *FvNAC28* and *FvNAC29* being up-regulated, and *FvNAC09*, *FvNAC18* and *FvNAC20* being down-regulated. As shown in Fig 13 (S14 Table), *FvNAC* genes (*FvNAC-02*, *-13*, *-14*, *-21*, *-26*, and *-32*) were steadily expressed under *R*. *solanacearum* infection and were found to be down-regulated, while no *FvNAC* genes were steadily up-regulated. However, *FvNAC01*, *-03*, *-04*, *-18*, *-28*, *-29*, *-34*, *36* and *-37* tended to be up-regulated (value>10-fold) at 1 d post-infection.

## Discussion

Woodland strawberries face severe destruction from various abiotic and biotic stresses (particularly *C*. *gloeosporioides* and *R*. *solanacearum*) during the growth and developmental stages. In order to address this, farmers and researchers have been increasing the cultivation area and improving cultivated techniques. However, the effects of this appear to be very limited [62,63]. Considering the common involvement of NACs in the plant stress response, *FvNACs* were chosen as candidate genes for further investigation for their potential use in genetic breeding in woodland strawberries. Overall, 37 *FvNACs* were identified from across the genome and the phylogenetic evolution of these genes was also revealed. Using quantitative real-time PCR analysis, the comprehensive expression profiles of the 37 *FvNACs* were assessed. To our knowledge, this is the first study to extend our understanding of the *FvNAC* gene family. Generally, the expression profiles could be divided into 2 sections. The first involves the expression of 37 *FvNACs* at different developmental stages, or in different tissues, and comprises the basic information of this gene family. We discovered that the transcripts of some *FvNACs* have strong expression levels for specific tissue and developmental stage, indicating their possible roles in specific growth or developmental stages, such as in the flowers or fruit. The other section involves gene expression in response to various abiotic and biotic stresses, which intends to identify several candidate genes commonly regulated by various stresses for stress-related genetic breeding.

In this study, multiple abiotic and biotic stress-responsive *FvNACs* were identified, and were in accordance with previous studies of the NAC gene family in other plant species, including *Arabidopsis* [7], *O*. *sativa* [7], *Nicotiana tabacum* [37], *P*. *trichocarpa* [38], *V*. *vinifera* [39], *S*. *italic* [40], *G*. *raimondii* [41], *M*. *acuminate* [42], *C*. *arietinum* [43], *M*. *esculenta* [44], *Z*. *mays* [45], *M*. *notabilis* [46], *C*. *melo* [47], and *S*. *lycopersicum* [48].

*ANAC042*, which showed a high degree of similarity with *FvNAC35*, was previously reported to be involved in the regulation of camalexin biosynthesis, and oxidative and heat stress resistance [24,32]. *FvNAC28* shared high similarity with *ATAF1*/*ANAC002*, which was previously shown to be involved in abiotic (drought, salt, and ABA) and biotic (necrotrophic pathogen *B*. *cinerea*) stress responses [8]. These results provide firm evidence of the protective roles of plant NACs in abiotic and biotic stress responses.

Cold stress is a major environmental factor affecting crop productivity and plant growth, development [64]. In *Arabidopsis*, *ANAC053* and *ANAC017* were found to be induced by cold stress [65]. In rice, 16 NAC genes (*ONAC007*, *-010*, *-015*, *-027*, *-028*, *-039*, *-045*, *-059*, *-067*, *-068*, *-073*, *-074*, *-085*, *-103*, *-122* and *-132*) showed up-regulation under cold treatment [66]. In our study, under cold treatment following recovery, *FvNAC03* and *FvNAC31* were up-regulated at all treated time points, and constituted the most highly induced genes (over 15-fold at 4 time points, respectively; Fig 4, S5 Table).

In tea (*Camellia sinensis*) plants, 7 *CsNAC* genes (*CsNAC02*, *CsNAC17*, *CsNAC26*, *CsNAC29*, *CsNAC30*, and *CsNAC32*) showed up-regulation under heat (40°C) stress at 24 h [67]. Similarly, *FvNAC15* and *FvNAC31* were up-regulated at 2 h, and *FvNAC36* between 2 d and 14 d, constituting the most highly induced genes (over 15-fold; Fig 5, S6 Table).

Under drought treatment, *FvNAC36* showed significant induction between 2 h and 3 d (Fig 6, S7 Table). In *Arabidopsis*, rice, maize, chickpea, and apple, 6 *ANAC* genes [19,20,68,69], 4 *OsNAC* genes [22,23,49,21,70], 8 *ZmNAC* genes [71], 14 *CaNAC* genes [43], and 12 *MdNAC* genes [72], were up-regulated under drought treatment, respectively.

Furthermore, under salt treatment, *FvNAC04* and *FvNAC29* showed significant up-regulation at all treated time points (Fig 7, S8 Table). In *Arabidopsis*, 10 *ANAC* genes [8,15,19,73–78], 21 *OsNAC* genes [21,66,79], 4 *CsNAC* genes [67], and 8 *MdNAC* genes [72] were up-regulated under drought treatment. These studies indicate that NAC family genes may be positively involved in the salt stress response.

Under H_2_O_2_ treatment, *FvNAC03*, *FvNAC04*, and *FvNAC29* showed significant up-regulation at all treated time points (Fig 8, S9 Table). In *Arabidopsis*, some evidence suggests that *NAC* genes, including *ANAC013*, *ANAC042*, and *ANAC059*/*ORS1*, play a positive role in the response to oxidative stress.

Under ABA treatment, *FvNAC30* tended to be up-regulated at all treated time points. In addition to *FvNAC18*, *FvNAC20*, and *FvNAC23*, all *FvNAC* genes tended to be up-regulated at the 1 d time point (over 2-fold; Fig 9, S10 Table). In *Arabidopsis*, several *ANAC* genes have been shown to regulate ABA mediated processes [14,69,73,77,80–83]. Among them, *FvNAC30* and *ANAC010*, *FvNAC28* and *ATAF1*/*ANAC002*, *FvNAC17* and *ANAC012*, and *FvNAC28* and *ATAF1*/*ANAC002* were found to be closely related and orthologous. Furthermore, the rice gene *OsNAC19* has been shown to regulate ABA mediated processes [34]. However, *FvNAC22* and *OsNAC19* were found to be closely related orthologs. The response of woodland strawberry NAC genes to ABA treatment suggested a possible role for *FvNAC* genes in ABA signaling.

Melatonin is a widely investigated, endogenously produced molecule in all plant species [84]. Previous reports have demonstrated the protective effects of melatonin to alleviate biotic and abiotic stresses [84]. *FvNAC03*, *FvNAC24*, *FvNAC29*, *FvNAC30* at 2 h, *FvNAC03*, *FvNAC27*, *FvNAC31* at 12 h, and *FvNAC24* at 1 d constituted the most highly induced genes (over 10-fold). On the contrary, 18 *FvNAC* genes under melatonin stress were found to be down-regulated (Fig 10, S11 Table). Thus, *FvNAC* family genes may be positively or negatively involved in the melatonin stress response.

Rapamycin, as an antifungal agent against pathogenic fungi, plays a crucial role in plant growth and metabolism [85–89]. Under rapamycin treatment, *FvNAC04* was up-regulated at all treated time points, and *FvNAC03* and *FvNAC28* tended to be up-regulated at 1 h to 2 d. However, 7 *FvNAC* genes were found to be down-regulated at all treated time points (Fig 11, S12 Table).

Plant NACs are involved in plant-pathogen interactions [25,29,31,90–92]. The *Arabidopsis ATAF1* is a regulator of the defense response against *B*. *cinerea*, *Blumeria graminis* f.sp. *hordei*, and *P*. *syringae* pv. *Tomato* [26]. Additionally, *HvSNAC1* has been found to be important for the resistance of barley inoculated with *Ramularia* leaf spot [93]. Under *C*. *gloeosporioides* infection, *FvNAC04*, *FvNAC28*, and *FvNAC29* tended to be up-regulated (Fig 12, S13 Table); meanwhile, under *R*. *solanacearum* infection, *FvNAC01*, *-03*, *-04*, *-18*, *-28*, *-29*, *-34*, *-36*, and *-37* tended to be up-regulated (over 10-fold) at 24 h (Fig 13, S14 Table). Moreover, *ATAF1* and *FvNAC28* were found to be closely related orthologs. This suggests that *FvNAC28* exhibits a similar function to *ATAF1*. Therefore, the NACs in the woodland strawberry are regulators of the defense response against *C*. *gloeosporioides* and *R*. *solanacearum*.

In conclusion, this study is the first to examine the *FvNAC* gene family along with their specific expression profiles, which may be used as potential candidates for further studies to dissect the function of *FvNAC* in response to stress stimuli.

The gene expression profiles obtained during 4°C, 40°C, drought, salt, H_2_O_2_, ABA, melatonin, rapamycin, *C*. *gloeosporioides* and *R*. *solanacearum* infection, and phytohormone treatments suggest that several woodland strawberry *NAC* genes could play important roles in environmental stress adaptation.

## Supporting information

S1 Table Oligonucleotide primers of 37 *FvNACs*. (XLS)

S2 Table Comprehensive identification of 37 *FvNACs*. (XLS)

S3 Table Accession numbers of NACs in *F*. *vesca*, *Arabidopsis*, and rice. (XLS)

S4 Table Expression data of the woodland strawberry *NAC* genes of different organs. (XLS)

S5 Table Expression data of the woodland strawberry *NAC* genes after cold stress. (XLS)

S6 Table Expression data of the woodland strawberry *NAC* genes after heat stress. (XLS)

S7 Table Expression data of the woodland strawberry *NAC* genes after drought stress. (XLS)

S8 Table Expression data of the woodland strawberry *NAC* genes after salt stress. (XLS)

S9 Table Expression data of the woodland strawberry *NAC* genes after H2O2 stress. (XLS)

S10 Table Expression data of the woodland strawberry *NAC* genes after ABA stress. (XLS)

S11 Table Expression data of the woodland strawberry *NAC* genes after melatonin stress. (XLS)

S12 Table Expression data of the woodland strawberry *NAC* genes after rapamycin stress. (XLS)

S13 Table Expression data of the woodland strawberry *NAC* genes after *C*. *gloeosporioides* infection. (XLS)

S14 Table Expression data of the woodland strawberry *NAC* genes after *R*. *solanacearum* infection. (XLS)

S1 Fig Conserved amino acid motifs of *FvNACs*. Conserved motifs and the sequence logos were generated using the MEME search tool. Numbers on the horizontal axis represent the sequence positions in the motifs and the vertical axis represent the information content measured in bits.
(TIF)
