# Supplementary figures and images for: Genome-wide identification and expression profile analysis of the NAC transcription factor family during abiotic and biotic stress in woodland strawberry
Source: PLoS One. 2018 Jun 13;13(6):e0197892. doi: 10.1371/journal.pone.0197892 (PMC5999216; doi:10.1371/journal.pone.0197892)

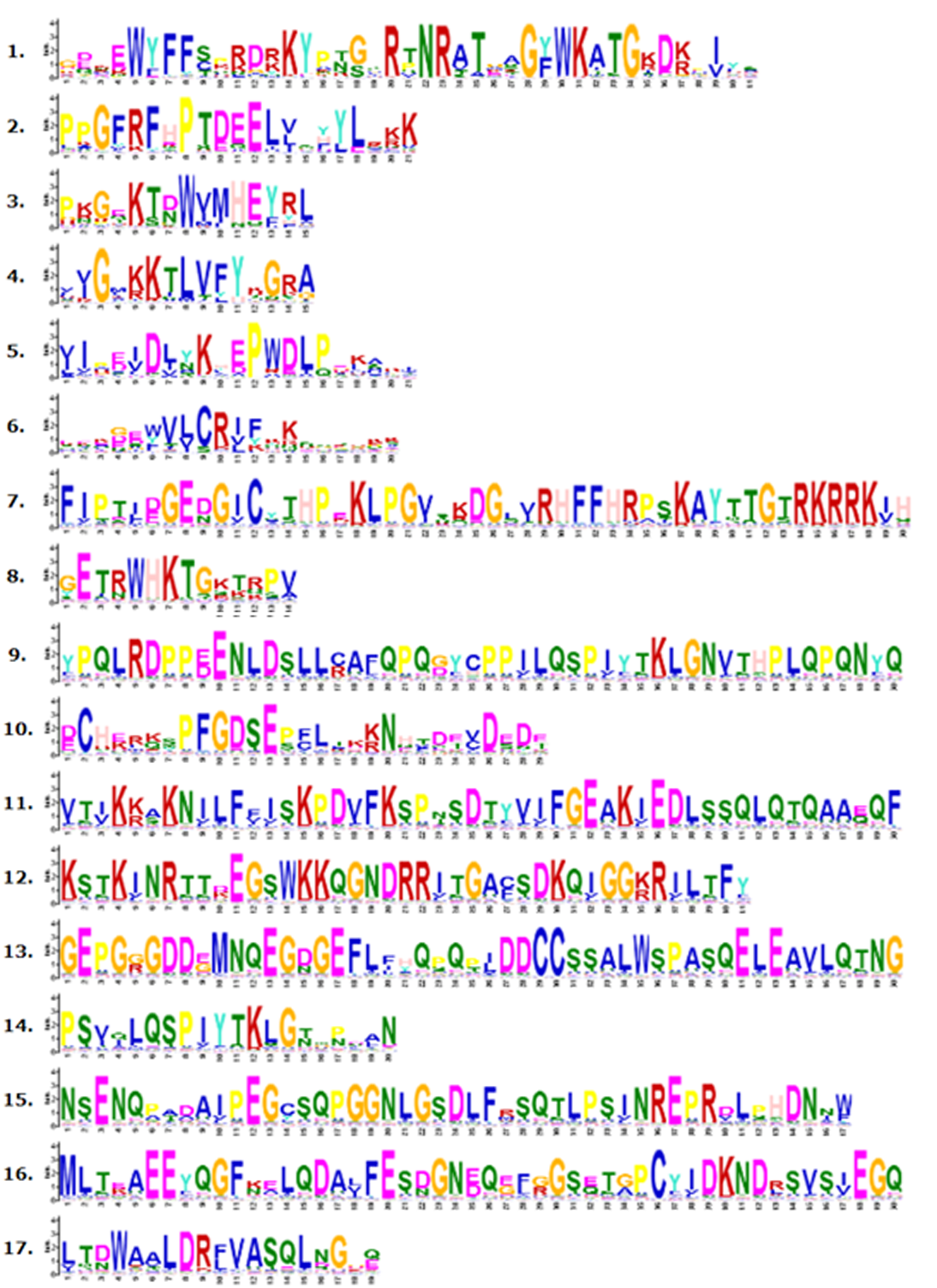

Supplement: S1 Fig — Conserved motifs and the sequence logos were generated using the MEME search tool. Numbers on the horizontal axis represent the sequence positions in the motifs and the vertical axis represent the information content measured in bits. (TIF) [file pone.0197892.s015.tif]
